# Specific alterations of gut microbiota in patients with membranous nephropathy: A systematic review and meta-analysis

**DOI:** 10.3389/fphys.2022.909491

**Published:** 2022-11-01

**Authors:** Yumeng Zhang, Jin Zhao, Yunlong Qin, Yuwei Wang, Zixian Yu, Xiaoxuan Ning, Shiren Sun

**Affiliations:** ^1^ Department of Postgraduate Student, Xi’an Medical University, Xi’an, China; ^2^ Department of Nephrology, Xijing Hospital, Fourth Military Medical University, Xi’an, Shaanxi, China; ^3^ Department of Nephrology, 980th Hospital of People’s Liberation Army Joint Logistic Support Force (Bethune International Peace Hospital), Shijiazhuang, Hebei, China; ^4^ Department of Geriatric, Xijing Hospital, Fourth Military Medical University, Xi’an, Shaanxi, China

**Keywords:** gut microbiota, membranous nephropathy, kidney disease, meta-analysis, systematic review

## Abstract

**Background:** The pathogenesis of idiopathic membranous nephropathy (IMN) has not yet been thoroughly clarified, and gut dysbiosis may be a contributor to IMN. However, the characterization of gut microbiota in patients with IMN remains uncertain.

**Methods:** Cochrane Library, PubMed, China National Knowledge Internet, Web of Science, and Embase were used to search for studies through 18 May 2022. A meta-analysis based on the standardized mean difference (SMD) with 95% confidence interval (CI) was conducted on the alpha diversity index. The between-group comparison of the relative abundance of gut microbiota taxa and the beta diversity were extracted and qualitatively analyzed.

**Results:** Five studies were included involving 290 patients with IMN, 100 healthy controls (HCs), and 129 patients with diabetic kidney disease (DKD). The quantitative combination of alpha diversity indices indicated that although bacterial richness was impaired [ACE, SMD = 0.12, (−0.28, 0.52), *p* = 0.55, *I* ^*2*^ = 0%; Chao1, SMD = −0.34, (−0.62, −0.06), *p* < 0.05, *I* ^*2*^ = 36%], overall diversity was preserved [Shannon, SMD = −0.16, (−0.64, 0.31), *p* = 0.50, *I* ^*2*^ = 53%; Simpson, SMD = 0.27, (−0.08, 0.61), *p* = 0.13, *I* ^*2*^ = 0%]. The beta diversity was significantly varied compared to HCs or DKD patients. Compared to HCs, the abundance of *Proteobacteria* increased, while that of *Firmicutes* decreased at the phylum level. Furthermore, the abundance of *Lachnospira* were depleted, while those of *Streptococcus* were enriched at the genus level. *Proteobacteria* and *Streptococcus* were also increased compared to DKD patients.

**Conclusions:** The expansion of *Proteobacteria* and depletion of *Lachnospira* may be critical features of the altered gut microbiota in patients with IMN. This condition may play an important role in the pathogenesis of IMN and could provide bacterial targets for diagnosis and therapy.

## Introduction

Idiopathic membranous nephropathy (IMN) is one of the most common pathological types of chronic kidney disease (CKD) and nephrotic syndrome in adults and is an immune-mediated glomerular disease characterized by the deposition of numerous immune complexes on the epithelial side of the glomerular capillary loops (Ronco et al., 2021). Currently, the pathogenesis of IMN is principally accepted to involve a combination of the target antigen of glomerular podocytes combined with autoantibodies to form immune complexes, triggering complement that causes tissue damage; but the mechanism has not been sufficiently clarified (Ronco and Debiec, 2020). The targeted antigens identified thus far include the M-type phospholipase A2 receptor (PLA2R), the thrombospondin type 1 domain-containing 7A (THSD7A), exostosin 1/exostosin 2 (EXT1/EXT2), the neural EGF-like-1 protein (NELL-1), semaphorin 3B (Sema3B), protocadherin 7 (PCDH7), and the high-temperature requirement A serine peptidase 1 (HTRA1), which all bind to antibodies of the IgG subtype (Sethi, 2021). The existing therapy for IMN primarily incorporates symptomatic treatment and etiological treatment including corticosteroids, immunosuppressive drugs and rituximab. While this treatment maintains a satisfactory prognosis for most patients (Couser, 2017), there are still almost 30% whose symptoms are not alleviated. Thus, it is urgent to explore the precise mechanisms of IMN. Recent studies have shown that gut dysbiosis may play a vital role in the pathogenesis of CKD (Zhao J. et al., 2021).

A vast ecosystem of microbes lives in the human gut, which has coevolved with humans for mutual benefit (Davenport et al., 2017), in preserving health and preventing disease (Ramezani and Raj, 2014). According to the “gut-kidney axis” theory, the disturbance of gut microbiota is closely related to CKD and interacts as both cause and effect. The gut microbes can activate immune cells via their metabolites and other components, causing inflammatory reactions and accelerating the advancement of kidney disease (Pahl and Vaziri, 2015). Antigens derived from the gut microbial community can induce conventional T cells to differentiate into a wide range of effector cells such as Th2 cells, Th17 cells, and T regulatory cells (Tregs) (Ivanov et al., 2022), which regulate the autoimmune response and immune homeostasis, thus affecting the occurrence and development of IMN (Cremoni et al., 2020; Zhao Q. et al., 2021). Systematic exposure to different microbial populations significantly stimulated B cells and induced the production of characteristic immunoglobulins such as IgG (Li et al., 2020), which may bind to IMN target antigens to mediate kidney damage. Our previous study reported that gut dysbiosis was a hallmark of patients with IMN (Dong et al., 2020) and contributed to high morbidity. Thus, we postulated that immune disorders regulated by gut microbiota dysbiosis under abnormal pathological conditions may initiate autoimmune diseases, including IMN. Secreted PLA2-IIA has been found to influence the immune system by hydrolyzing bacterial membranes and altering the composition of gut microbiota. It participates in the induction of immunophenotypes and promotes the inflammatory response (Dore et al., 2022), suggesting a potential relationship between the PLA2R antigen of IMN and gut dysbiosis. Currently, modulation of gut dysbiosis via fecal microbiota transplantation (FMT) has been shown to have promising efficacy for treating patients with IMN (Zhou et al., 2021).

The abovementioned results suggest that gut dysbiosis may be tightly correlated with IMN and is conceivably a critical target for treatment of IMN. However, the conclusions conveyed in the literature on the traits of gut microbiota in patients with IMN deviate extensively and the characteristic features are unclear. Therefore, we conducted a meta-analysis of data on the alterations in gut microbiota alpha diversity. A qualitative synthesis of the relative abundance of gut microbial taxa at different levels was also performed and beta diversity was determined. Specific metabolic characteristics of patients with IMN were assessed to identify the essential markers of gut microbiota and to provide new insights for the prevention and treatment of IMN.

## Materials and methods

### Literature retrieval and quality evaluation

We followed the guidelines of the preferred reporting project in the systematic review and meta-analysis (PRISMA), and the study was registered in the PROSPERO database (CRD42022307282). The combination of free words of medical subject terms (MeSH) or keywords, including all spellings of MN, gut, stool, and microbiota (Supplementary Table S1), were used to search for studies on gut microbiota of patients with IMN in the Cochrane Library, PubMed, China National Knowledge Internet, Web of Science, and Embase. The retrieval period was from the database creation time to 18 May 2022. To be included, a report had to be on a cross-sectional study or a case-control study and the study subjects had to be patients with IMN confirmed by clinicopathological examination. The exclusion criteria were: no control group established, unrelated confounding factors, repeated publications, inability to obtain the original data or to contact the author to obtain the original data, the abstract, the review, meta-analysis, case reports, or animal experiments. The coauthors, Zhang Y and Zhao J, screened the title, abstract, and full text of the articles to ensure consistency. The quality of the included case-control studies was assessed using the Newcastle–Ottawa scale (NOS) (Stang, 2010). The scale consisted of eight items and evaluated the three dimensions of selectivity, comparability, and exposure factors. The score, ranging from 0 to 10, indicated the quality of the publication.

### Data extraction

The following information was extracted from each study: characteristics, analysis methods, sample collection, alpha diversity indices (the maximum, minimum, mean, M, and standard deviation, SD, of the ACE, Chao1, Shannon, and Simpson indices), beta diversity, differential taxa, and functional features of the gut microbiota. The alpha diversity indices were used to estimate the community richness (ACE and Chao1) and community diversity (Shannon and Simpson), while beta diversity was applied to measure the rate of change of the species diversity between communities. Excel was used to record and organize the data extracted from the selected studies. For parameters with quantitative descriptions including alpha diversity indices, if the original data only provided median and quartile range, a Web-based tool (http://www.math.hkbu.edu.hk/∼tongt/papers/median2mean.html) was used to convert it to mean and standard deviation. WebPlotDigitizer (Drevon et al., 2017) was used to extract digital data from figures if necessary. The two researchers (Zhang Y and Zhao J) independently extracted literature information, and any disputed content was resolved through discussion and negotiation by a third researcher (Sun S).

### Quantitative synthesis

Meta-analysis of the alpha diversity indices reported from two or more studies was performed using RevMan5.3 software provided by the Cochrane collaboration, and the standardized mean difference (SMD) between the IMN patient group and the controls was calculated to analyze the differences in alpha diversity between the two groups. The *I* ^*2*^ test was used to test the heterogeneity of the included studies. If *I* ^*2*^ was ≤ 50%, the heterogeneity was small and a fixed-effect model was used for analysis; if *I* ^*2*^ was >50%, the heterogeneity was high and a random effects model was adopted for analysis (Patrick et al., 2020). If the *p* values on the two sides were < 0.05, the differences were considered to be statistically significant.

### Qualitative synthesis

The beta diversity of the microbiota and the relative abundance of its different constituents at the phylum and genus levels were qualitatively synthesized. To aid in data interpretation, we defined a parameter that the results were specific if they changed in the same direction in more than two studies and no study showed contrary results (Zhao J. et al., 2021). Results reported by only one study were excluded, because such results are considered potentially methodological- or population-specific, and thus may be false-positive results.

## Results

### Search results and characteristics

**FIGURE 1:**
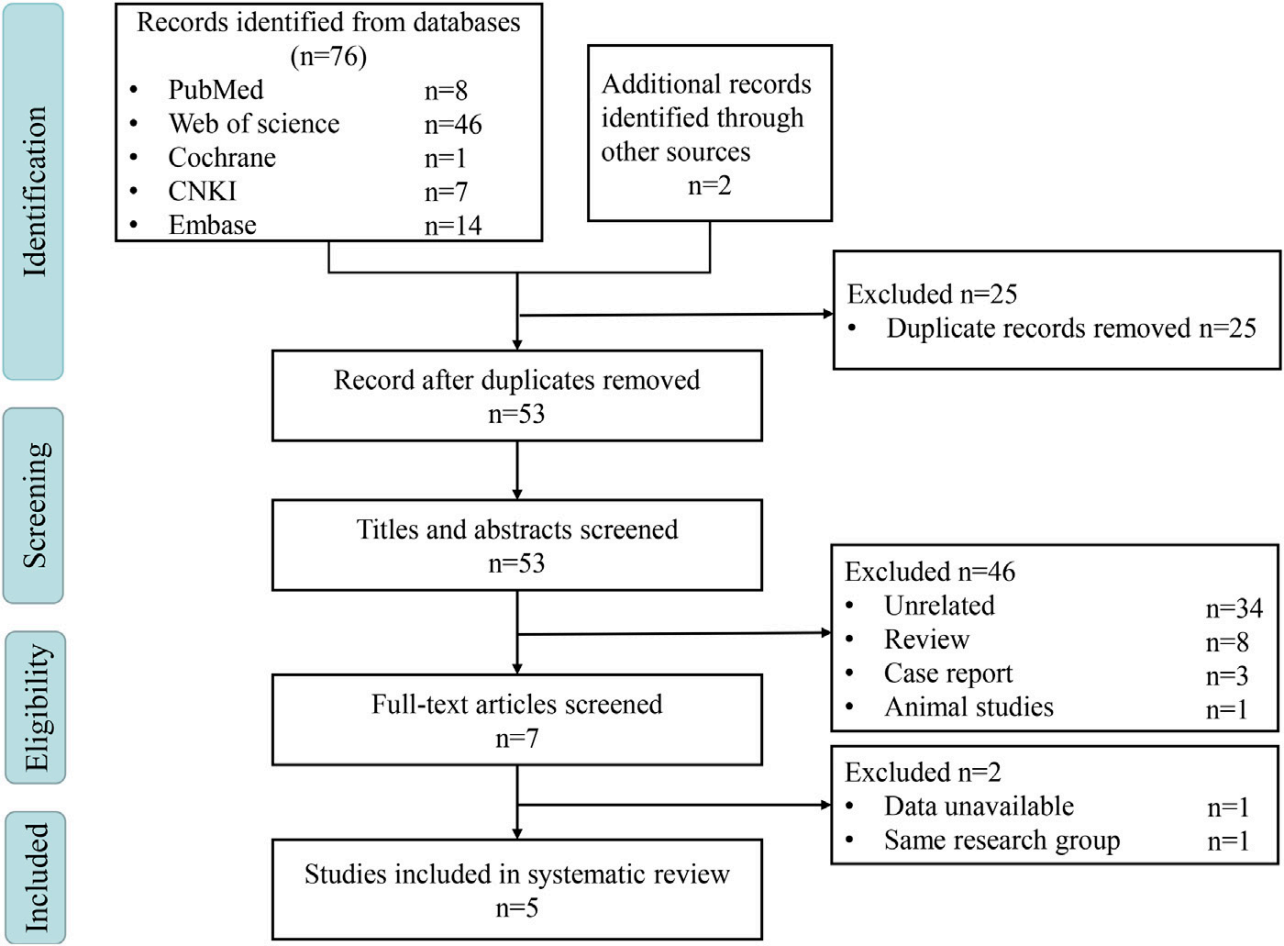
PRISMA flow chart. CNKI, China National Knowledge Internet.

A total of 78 potentially eligible articles were obtained from the literature search, and five studies (Dong et al., 2020; Lang et al., 2020; Yu et al., 2020; Zhang et al., 2020; Li et al., 2022) were eventually included (Figure 1). All studies were of the case-control type, with four studies using healthy individuals as controls (HCs) and one study using patients with diabetic kidney disease (DKD) as the control group. The study researchers collected and analyzed stool samples from 519 participants from China, and16S rRNA gene sequencing was used to characterize the gut microbiota. A summary of the literature features is shown in Table 1. Additionally, all five studies mentioned freezing conditions for sample transfer or storage, while only two studies mentioned the use of special sample collection containers (Supplementary Table S2). The NOS scale was used to evaluate the quality of the reports, and four of the studies had an evaluation score of six* while the fifth was five* (Supplementary Table S3), which confirmed that the reports were of high quality.

**TABLE 1:** Characteristics of studies included in this review.

References	Year	Region	Study design	Center number	Subject sample, *N*	Age in years	Basic conclusions
Wei Yu et al.	2020	Zhengzhou, China	Case-control study	1	*N* = 271	DKD 56.5 ± 3.1	The composition of the gut microbiome appears to differ considerably between patients with DKD and those with MN.
					DKD (*n* = 129)	IMN 49.2 ± 2.5	
					IMN (*n* = 142)		
Ruijuan Dong et al.	2020	Xi’an, China	Case-control study	1	*N* = 70	HCs 38.6 ± 12.8	Patients with MN exhibited gut microbial signatures distinct from healthy controls.
					HCs (*n* = 30)	IMN 43.1 ± 13.8	
					IMN (*n* = 40)		
Jun Zhang et al.	2020	Guangdong, China	Case-control study	1	*N* = 78	HCs 46.5 ± 22.7	MN patients had significantly different alpha and beta diversity and decreased gut microbiota-derived short-chain fatty acids.
					HCs (*n* = 30)	IMN 48.5 ± 20.5	
					IMN (*n* = 48)		
Rui Lang et al.	2020	Beijing, China	Case-control study	1	*N* = 40	HCs 44.1 ± 9.2	Patients with IMN might have disordered intestinal microbiota.
					HCs (*n* = 10)	IMN 48.8 ± 11.3	
					IMN (*n* = 30)		
Mengfei Li et al.	2022	Heilongjiang, China	Case-control study	1	*N* = 60	HCs 45.2 ± 13.2	Patients with IMN appear to have an altered gut microbiome.
					HCs (*n* = 30)	IMN 51.3 ± 10.3	
					IMN (*n* = 30)		

DKD, diabetic kidney disease; MN, membranous nephropathy; IMN, idiopathic membranous nephropathy; HCs, healthy controls.

### Alpha diversity

**FIGURE 2:**
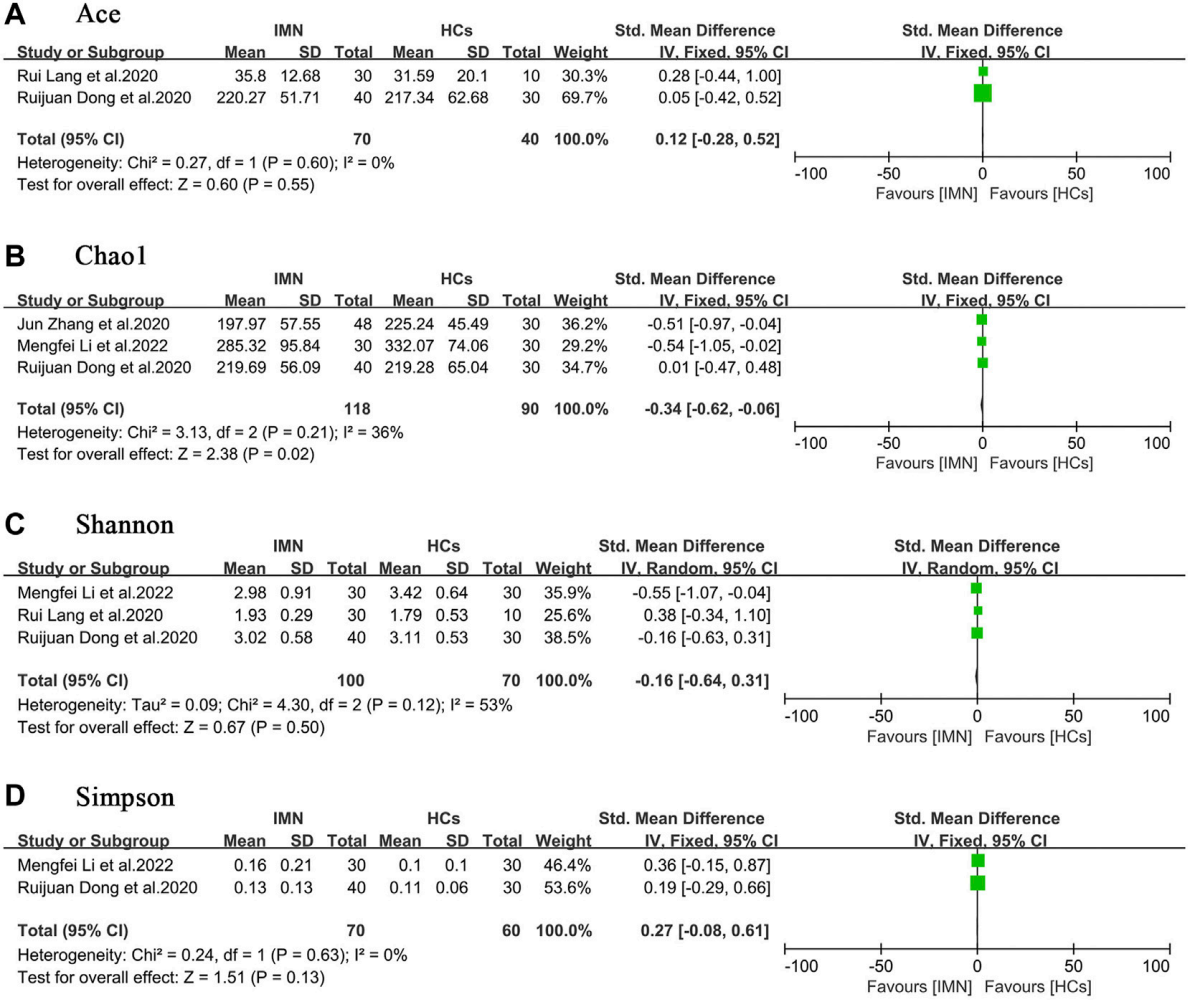
Forest plots of alpha diversity in the gut microbiota of patients with IMN compared with HCs. **(A)** Ace index, SMD = 0.12, [−0.28, 0.52]; *p* = 0.55; *I* ^*2*^ = 0%; **(B)** Chao1 index, SMD = −0.34, [−0.62, −0.06]; *p* < 0.05; *I* ^*2*^ = 36%; **(C)** Shannon index, SMD = −0.16, [−0.64, 0.31]; *p* = 0.50; *I* ^*2*^ = 53%; **(D)** Simpson index, SMD = 0.27, [−0.08, 0.61]; *p* = 0.13; *I* ^*2*^ = 0%. IMN, idiopathic membranous nephropathy; HCs, healthy controls.

Four studies compared the alpha diversity of gut microbiota in patients with IMN to that in HCs (Dong et al., 2020; Lang et al., 2020; Zhang et al., 2020; Li et al., 2022). In terms of richness, two studies (Dong et al., 2020; Lang et al., 2020) provided ACE index data, and three studies (Dong et al., 2020; Zhang et al., 2020; Li et al., 2022) provided Chao1 index data. In terms of diversity, three studies (Dong et al., 2020; Lang et al., 2020; Li et al., 2022) provided Shannon index data, and two studies provided Simpson index data (Dong et al., 2020; Li et al., 2022). The quantitative combination of alpha diversity indices indicated that although bacterial richness was impaired [ACE, SMD = 0.12, (−0.28, 0.52), *p* = 0.55, *I* ^*2*^ = 0%; Chao1, SMD = −0.34, (−0.62, −0.06), *p* < 0.05, *I* ^*2*^ = 36%], diversity was preserved overall [Shannon, SMD = −0.16, (−0.64, 0.31), *p* = 0.50, *I* ^*2*^ = 53%; Simpson, SMD = 0.27, (−0.08, 0.61), *p* = 0.13, *I* ^*2*^ = 0%] (Figure 2). One study (Yu et al., 2020) analyzed the alpha diversity of gut microbiota in patients with IMN or DKD, and because of the paucity of data, quantitative analysis was not possible.

### Beta diversity

In all five studies (Dong et al., 2020; Lang et al., 2020; Yu et al., 2020; Zhang et al., 2020; Li et al., 2022), the beta diversity of the gut microbiota was measured by principal coordinate analysis (PCoA) or principal component analysis (PCA) (Supplementary Table S5). The four studies (Dong et al., 2020; Lang et al., 2020; Zhang et al., 2020; Li et al., 2022) with HCs uncovered marked dissimilarities in the beta diversity of gut microbiota between patients with IMN and HCs. One study (Yu et al., 2020) demonstrated that the beta diversity in patients with IMN was also greatly altered compared to DKD patients.

### Differences in the abundance of bacterial taxa

All studies (Dong et al., 2020; Lang et al., 2020; Yu et al., 2020; Zhang et al., 2020; Li et al., 2022) analyzed the distinct taxa at the phylum level. Compared with HCs, three studies reported that the abundance of *Proteobacteria* increased (Dong et al., 2020; Zhang et al., 2020; Li et al., 2022) while that of *Firmicutes* decreased (Lang et al., 2020; Zhang et al., 2020; Li et al., 2022), respectively. The abundance of *Proteobacteria* also increased in IMN patients compared to DKD patients (Yu et al., 2020). The enrichment of *Actinobacteria* was observed in patients with IMN compared with HCs (Li et al., 2022) and in one group of DKD patients (Yu et al., 2020). The variations in the abundance of *Bacteroidetes* were inconsistent; it was increased in one study (Lang et al., 2020), but decreased in two studies (Yu et al., 2020; Li et al., 2022) (Supplementary Table S6).

In all studies (Dong et al., 2020; Lang et al., 2020; Yu et al., 2020; Zhang et al., 2020; Li et al., 2022), the alteration of gut microbiota was evaluated at the genus level. Compared with HCs, two studies indicated that the abundance of *Streptococcus* was increased in IMN (Dong et al., 2020; Li et al., 2022), and three studies reported that the abundance of *Lachnospira* was decreased (Dong et al., 2020; Zhang et al., 2020; Li et al., 2022). One study also discovered that *Streptococcus* was similarly amplified when compared with DKD patients (Yu et al., 2020). The abundance of *Peptostreptococcaceae_incertae_sedis* was enriched in IMN in one study compared with HCs (Dong et al., 2020), and was also expanded when compared with DKD patients (Yu et al., 2020)). In addition, the abundance of *Clostridium_sensu_stricto_1* (Dong et al., 2020), *Veillonella* (Dong et al., 2020), and *Faecalibacterium* (Li et al., 2022) was reduced in IMN patients compared with HCs, while it was increased compared to DKD patients (Yu et al., 2020) (Supplementary Table S7).

**FIGURE 3:**
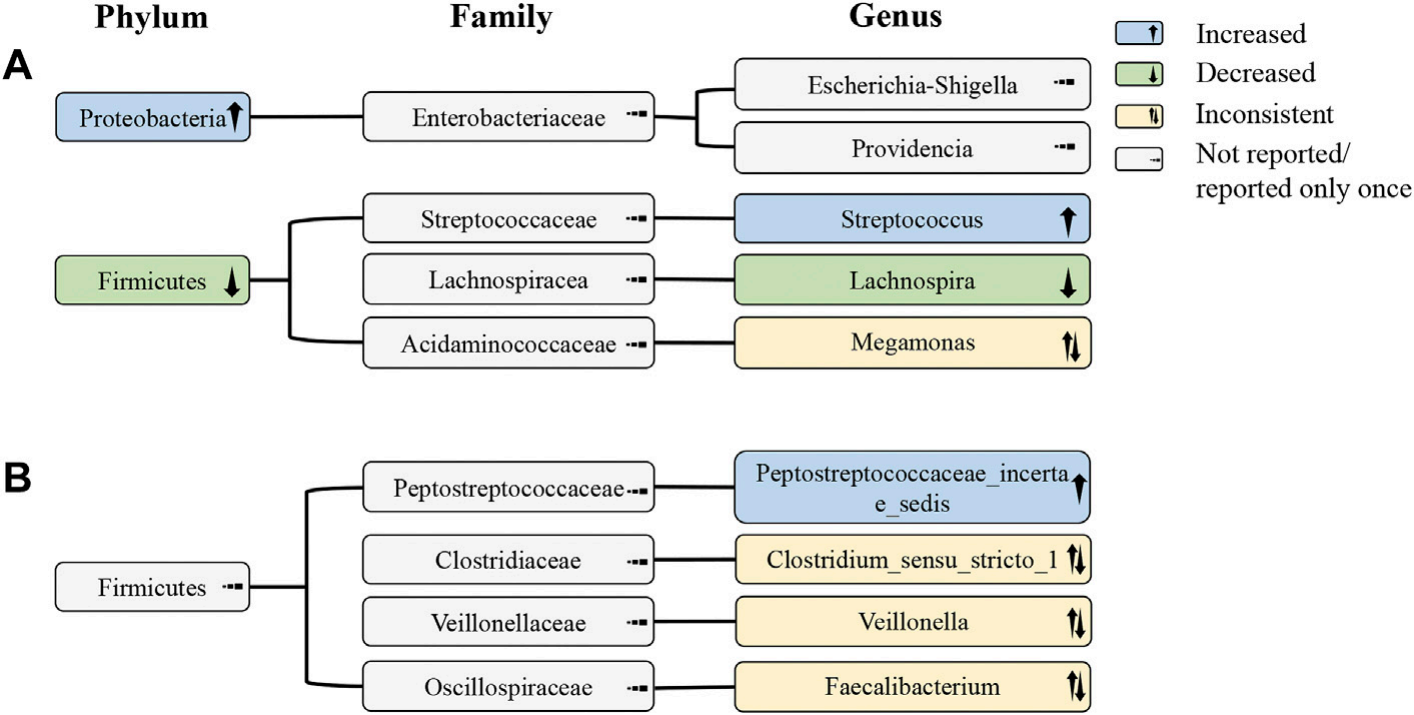
Alterations of gut microbiota at the phylum, family, and genus levels in IMN patients. **(A)** Alterations of gut microbiota in IMN patients compared with HCs; **(B)** alterations of gut microbiota in IMN patients relative to HCs and DKD. IMN, idiopathic membranous nephropathy; HCs, healthy controls; DKD, diabetic kidney disease.

According to the phylogenetic profile, the decrease in abundance of the genus *Lachnospira* was probably the most critical reason for the depletion of the phylum *Firmicutes*. The increase in *Streptococcus* did not reverse the decline in *Firmicutes*, suggesting that the change in *Streptococcus* may not be that large or that there may be a decrease in other undetected bacteria at the genus level. In addition, the abundance of the phylum *Proteobacteria* significantly increased, but no bacterial genus belonging to it with the same change was found (Figure 3).

### Metabolic characteristics of gut microbiota

Two studies (Yu et al., 2020; Li et al., 2022) analyzed the metabolic characteristics of the gut microbiota in patients with IMN (Supplementary Table S8). Wei Yu et al. (Yu et al., 2020) used LEfSe analysis to determine the crucial functional differences between DKD patients and patients with IMN according to the KEGG classifications, and found overexpression of membrane transporters involving the ABC transporters and the phosphotransferase system (PTS) in the microbiome of patients with IMN. Li et al. (Li et al., 2022) found that the KEGG pathway enrichment included alpha-linolenic acid metabolism, *Staphylococcus aureus* infection, and the arachidonic acid metabolic pathway in IMN patients compared to HCs. These results may represent functional differences in gut microbiota between IMN patients and healthy individuals.

## Discussion

To our knowledge, this is the first systematic review and meta-analysis to comprehensively analyze the profile of gut microbiota in patients with IMN and explore the characteristics of the gut microbiota that may be closely related to the occurrence and development of IMN. We observed that the results of the Chao1 index analysis, which represents community richness, showed meaningful distinctions in alpha diversity. Significant alterations in beta diversity of the gut microbiota were also observed in IMN patients. At the phylum level, the abundance of *Proteobacteria* increased, while that of *Firmicutes* decreased. At the genus level, the number of *Lachnospira* was depleted, while *Streptococcus* was enriched.

Alpha diversity is a commonly used metric in studies involving gut microbiota. Since reduced diversity is generally considered to be a manifestation of weakened health in the host or outright disease (Shade, 2017), we expected that patients with IMN would have reduced gut microbial diversity, as observed in many diseases (Scher et al., 2015). However, our study only showed reduction in the Chao1 index data for alpha diversity in patients with IMN, indicating that although the abundance of certain bacterial species was somewhat reduced, the overall diversity was preserved. Zhao H et al. (Zhao et al., 2021) analyzed the changes in gut microbiota alpha diversity in IMN rats and found that gut microbiota richness and diversity were both decreased, which is inconsistent with our results. If confounding factors such as specimen storage and testing methods are excluded, the differences in results may be related to differences in disease severity. Also, quantitative analysis may produce false-negative results due to the relatively low number of studies included. In gut microbiota, the beta diversity index is used to measure the rate of species diversity change along environmental gradients and intercommunity diversity. The significant alteration in beta diversity revealed that the gut microbial community in IMN patients was altered; therefore, we consider that gut dysbiosis was present in patients with IMN.

**FIGURE 4:**
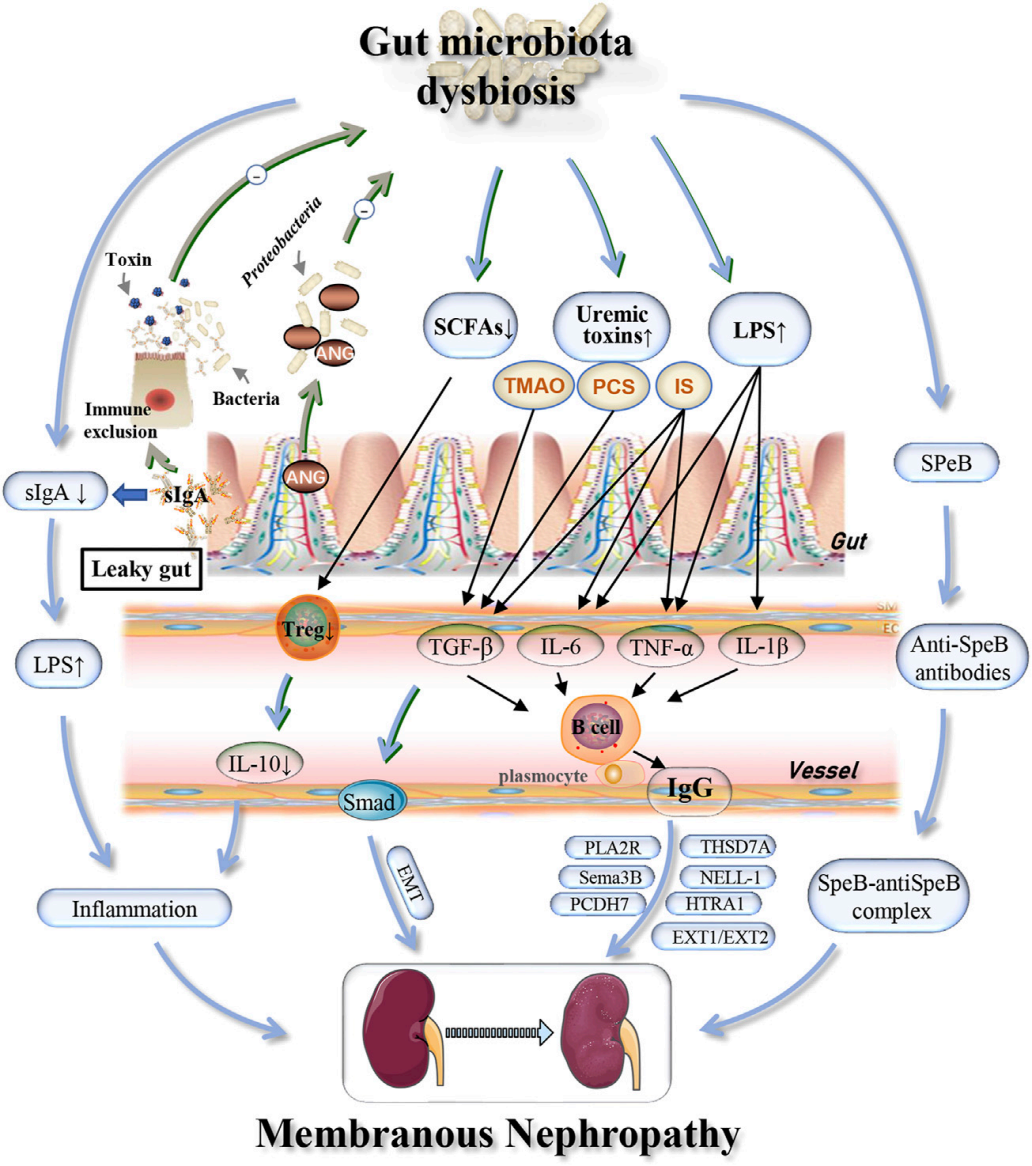
Possible mechanisms associated with gut dysbiosis and effects of host immunity on gut microbiota in IMN patients. Alterations in the specific microbiome may be involved in the pathogenesis and development of IMN through depletion of SCFAs and sIgA, increased cytokines (TNF-α, IL-1β), uremic toxins (PCS, IS, TMAO), and common antigens (SPeB) with the glomerular basement membrane. The host immune system can regulate the gut microbiota homeostasis through the production of sIgA and the induction of Ang. IMN, idiopathic membranous nephropathy; sIgA, secretory immunoglobulin A; TMAO, trimethylamine N-oxide; PCS, p-cresol sulfate; IS, indoxyl sulfate; IL, interleukin; TNF-α, tumor necrosis factor-α; TGF-β, transforming growth factor-β; Treg, regulatory T cells; EMT, epithelial-mesenchymal transition; SPeB, streptococcal pyrogenic exotoxin B; PLA2R, phospholipase A2 receptor; THSD7A, thrombospondin type 1 domain-containing 7A; EXT1/EXT2, exostosin 1/exostosin 2; NELL-1, neural EGF-like-1 protein; Sema3B, semaphorin 3B; PCDH7, protocadherin 7; HTRA1, high-temperature requirement A serine peptidase 1; Ang, angiogenin.

Further analysis revealed significant expansion of *Proteobacteria* in patients with IMN. A previous study found that *Proteobacteria* was also increased in patients with DKD (He et al., 2022). Thus, we believe that, as with DKD, the increase in *Proteobacteria* in IMN patients was meaningful and may indicate an intestinal inflammatory response and intestinal epithelial cell dysfunction (Litvak et al., 2017). The impaired intestinal epithelial function could cause further systemic inflammation, which would accelerate the development of kidney disease and result in more damage (Pahl and Vaziri, 2015). For example, lipopolysaccharide (LPS) derived from *Escherichia coli* (*E. coli*), which belongs to the phylum *Proteobacteria*, can induce the production of the cytokines tumor necrosis factor α (TNF-α), interleukin-1β (IL-1β), and interleukin-6 (IL-6) through the TLR4–NF-κB and TLR4–p38MAPK pathways, resulting in an inflammatory response (Qiu et al., 2021). Polymorphisms in the Toll-like receptor 4 (TLR4) gene and the IL-6 gene were associated with IMN morbidity (Liu et al., 2019). Moreover, genome-wide association studies (GWAS) in IMN patients revealed that a site on the NF-κB1 gene was associated with disease risk (Xie et al., 2020). Therefore, we speculated that *Proteobacteria* might be involved in the pathogenesis of IMN by upregulating expression of the TLR4, IL-6 and NF-κB1 genes. Secretory immunoglobulin A (sIgA), found in the intestinal mucosal immune barrier, is secreted by B cells induced by symbiotic microorganisms penetrating Peyer’s patches. This induction can produce a potent mucosal immune response including neutralizing endotoxins and enveloping bacteria to prevent pathogen invasion and maintain gut microbial homeostasis through the NF-κB pathway to reduce the inflammatory response (Pietrzak et al., 2020). By analyzing the correlation between gut microbiota and fecal sIgA in patients with early IMN, the change in *E. coli* may be linked abnormalities in sIgA, thus leading to an increase in intestinal permeability (Wang, 2017). Destruction of the intestinal mucosal barrier can result in the systemic circulation of bacteria and bacterial products and promote the accumulation of a large number of metabolic wastes in the body, especially indoxyl sulfate (IS), p-cresol sulfate (PCS) and trimethylamine oxide (TMAO) (Vaziri et al., 2016). IS can increase the secretion of TNF-α, IL-6 and other cytokines, thus stimulating B cells to produce IgG antibodies that participate in the pathogenesis of IMN (Liu et al., 2018). Moreover, IS, PCS and TMAO induced the expression of transforming growth factor β1 (TGF-β1) (Simona et al., 2017) and promoted the epithelial-mesenchymal transition (EMT) (Geng et al., 2020), leading to renal injury (Figure 4). Multiple cytokines, including TNF-α and IL-1β were also found to be elevated in untreated celiac patients compared with HCs and treated patients (Boonpheng et al., 2018). Celiac disease is an inflammatory disease of the small intestine, and renal insufficiency, including membranous nephropathy, is one of its known manifestations (Hamla and Ubels, 2007). Tacrolimus is a commonly used drug for the treatment of IMN. It interferes with T cell-induced activation of TNF-α, IL-1β and IL-6 by inhibiting calcineurin, thereby blocking the immune response and delaying the progression of renal injury (Tanaka et al., 2012). As a macrolide antibiotic, tacrolimus can also alter the gut microbiota, which is one of the possible mechanisms of its immunosuppressive effect. Further studies may focus on the prognosis of IMN by observing the effect of different treatment regimens on changes in gut dysbiosis, for monitoring efficacy and adjusting medication. *Salmonella* also belongs to the phylum *Proteobacteria* and can reduce the number of IgG-secreting plasma cells in the bone marrow, resulting in a decrease in serum IgG titer and immune complex formation (Manne et al., 2019). In summary, *Proteobacteria* is closely associated with IMN, but the mechanism remains unclear. As screening for *Proteobacteria* in patients with IMN at the genus level has not been reported thus far, more studies are needed to identify changes in *Proteobacteria* at the species level.

The abundance of the phylum *Firmicutes* and genus *Lachnospira* was significantly decreased in patients with IMN. A previous study with high-throughput sequencing of the fecal microbiome of CKD patients and HCs found that *Lachnospira* was significantly reduced in CKD patients, which is a good microbial marker (Lun et al., 2019). *Firmicutes* are bacteria that produce a large amount of butyric acid (Jandhyala et al., 2015), and *Lachnospira* is an obligate anaerobic bacterium that decomposes carbohydrates to produce short-chain fatty acids (SCFAs) (Vacca et al., 2020). Jun Zhang et al. (Zhang et al., 2020) analyzed fecal samples from IMN and control groups for organic acids and found that the SCFAs, propionic acid and butyric acid, were significantly lower in patients with IMN than in healthy controls. In addition, in a study analyzing the fecal flora of IMN rats, it was discovered that the numbers of bacteria producing butyric acid in the intestinal tract were lower than in control rats (Wang et al., 2022). These findings suggested that the decreased abundance of *Lachnospira* may result in lower production of SCFAs. Alteration of the Th17/Treg ratio is a possible mechanism for the pathogenesis of IMN (Motavalli et al., 2021), because SCFAs derived from microbial metabolites regulate Treg cell homeostasis, which has been proven to be critical for the health of gut microbiota and maintaining the integrity of the intestinal epithelial cells that inhibit invasion of gut microbiota (Furusawa et al., 2013). Treg cells were also found to be essential for autoantigen tolerance and the prevention of autoimmune diseases (Takahashi et al., 2020) Administration of SCFAs to mice can increase the differentiation of thymus-derived T-cells into peripheral Treg cells (Arpaia et al., 2013). Thus, lower production of SCFAs resulted in reduced production and differentiation of Treg cells, leading to lower production of the anti-inflammatory cytokine interleukin-10 (IL-10) and impaired anti-inflammatory effects (Proto et al., 2018) (Figure 4). The abovementioned results may be part of the mechanism by which *Lachnospira* is involved in the progression of IMN, and the intervention of *Lachnospira* and SCFAs may be a promising new direction in the treatment of IMN.

The induction of antimicrobial peptides (AMPs) is one of the mechanisms by which the host immune system establishes intestinal homeostasis of symbiotic microbes (Mami et al., 2016). As an AMP, angiogenin (Ang), secreted by Paneth cells, directly inhibits *α -Proteobacteria* strains by destroying the integrity of the bacterial membrane to balance the numbers of *α-Proteobacteria* and *Lachnospiraceae* and maintain homeostasis of the gut microbiota. Ang1 deficiency in mice resulted in expansion of *α-Proteobacteria* and reduction of protective intestinal symbiotic strains such as *Lachnospiraceae* (Sun et al., 2021). Ang1 is also an endothelial factor produced by podocytes and is involved in the regulation and maintenance of glomerular basement membrane (GBM) permeability (Satchell et al., 2004). Therefore, we hypothesized that damage to podocytes in IMN patients may reduce production of Ang1, resulting in characteristic disturbances of gut microbiota. Administration of Ang1 may be a promising intervention to treat IMN, although the mechanism of its action has not yet been reported, and further experiments are needed to test this hypothesis.

*Streptococcus* was observed to be elevated in patients with IMN but not HCs or DKD patients. Interestingly, an increased abundance of *Streptococcus* was also found in the salivary microbiota of IMN patients (Luan et al., 2022). LPS from *Streptococcus* can enter the blood through the damaged intestinal where it activates the NF-κB pathway and stabilizes hypoxia-inducible factor-1α, which triggers the release of inflammatory factors such as IL-6 and IL-1β, thus promoting the occurrence of inflammatory disease (Tannahill et al., 2013; Ramezani and Raj, 2014; Manfredo Vieira et al., 2018). IL-1β can promote Th17 cell responses (Park et al., 2017), which are involved in autoimmune diseases such as inflammatory bowel disease (IBD). It has been reported that the diversity and abundance of gut microbiota in IBD patients is highly consistent with the changes in gut microbiota in IMN patients (Nishida et al., 2018). Furthermore, streptococcal pyrogenic exotoxin B (SPeB) is a common antigen of the GBM, and antibodies generated by the body against *Streptococcus* may react with the GBM, resulting in deposition of immune complexes in the GBM and damage to the basement membrane (Balasubramanian and Marks, 2017). *Streptococcus* has also been significantly associated with a number of other kidney diseases. For example, *Streptococcus* was increased in Henoch-Schonlein purpura nephritis and disease severity was proportional to its abundance (Tan et al., 2022). Intravenous administration of *Streptococcus mutans* to rats can cause the transient induction of lesions similar to those from IgA nephropathy (Naka et al., 2020). Consequently, the amplification of *Streptococcus* may be involved in the progression of kidney injury through these possible mechanisms.

ABC transporters participate in the biogenesis of the outer membrane of gram-negative bacteria and mediate the separation of LPS from the inner membrane (Narita, 2011; Alexander et al., 2018). The stimulation of TLR4 by LPS derived from gram-negative bacteria induces the production of crucial pro-inflammatory cytokines and then activates the host immune response (Cochet and Peri, 2017). It was demonstrated that gut dysbiosis may be involved in IMN pathogenesis by inducing TLR4 gene expression. In another way, the expansion of ABC membrane transporters may mean an increase in bacteria such as *E. coli* that respond to LPS-mediated inflammation. More studies are needed to explore the metabolic function of gut bacteria in patients with IMN.

IMN is one of the most common pathological types of CKD, and its incidence has been increasing in recent years (Tang et al., 2017). In patients with CKD, membrane nephropathy is correlated with consistent changes in specific bacteria at the phylum and genus level, including an increase in *Proteobacteria* and *Streptococcus* and a reduction in *Firmicutes* and *Lachnospira* (Lun et al., 2019; Zhao J. et al., 2021). The abovementioned studies examined the changes in gut microbiota in patients with IMN, which may provide a more accurate basis for further studies to discover or verify specific bacterial taxa at the genus and species levels that are closely related to IMN. The identical results of the alteration in patients with CKD and IMN may be reasonable but the correlation is unclear. Further studies are needed to explore the changes in gut microbiota in patients with different pathological types of CKD to better determine the characteristic alterations of gut microbiota at the species level.

Microbiome-based precision medicine targeting specific alterations in gut microbiota in patients with IMN, including fecal microbial transplantation (FMT), prebiotics, probiotics, antibiotics, vaccines, and dietary immune stimulation products, may be a more selective and safer therapy for inhibiting the aberrant immune response and reducing kidney injury in patients with IMN. FMT has been applied to treat IMN patients and has shown promising efficacy, with alleviation of symptoms and improvement of renal function (Zhou et al., 2021). High-quality clinical studies are needed to validate the efficacy and potential mechanisms of microbial therapy in the future. In addition, whether gut dysbiosis involving specific strains are a cause or result of IMN remains undetermined, which warrants further investigation in well-designed interventional clinical trials or animal experiments.

This systematic review and meta-analysis still has some limitations. First, we did not evaluate publication bias due to the small number of included studies. Second, the selected studies did not distinguish the disease severity of patients, which needs to be addressed in future research. Third, the original studies were all performed in China, and caution must be exercised in applying these findings to non-Chinese populations. Therefore, high quality and multiregional, multicenter clinical studies are still needed to verify our findings.

## Conclusions

This review reveals that the beta diversity is changed and the richness is reduced in the gut microbiota of IMN patients. The alternation of gut microbiota structure in patients with IMN was characterized by augmentation of the phylum *Proteobacteria*, while depletion of butyric acid-producing bacteria mainly included the phylum *Firmicutes* and the genus *Lachnospira*. Notably, the genus *Lachnospira* was markedly depleted and, thus, may be closely related to the occurrence of IMN, which needs further exploration.

## Data Availability

All data obtained or scrutinized in this study is contained in this review and in Supplementary Material. Further queries can be addressed to the corresponding author.
