# Supplementary material for: Specific alterations of gut microbiota in patients with membranous nephropathy: A systematic review and meta-analysis
Source: Front Physiol. 2022 Nov 1;13:909491. doi: 10.3389/fphys.2022.909491 (PMC9664147; doi:10.3389/fphys.2022.909491)
Supplement: Supplementary file 1 [file DataSheet1.docx]

**Specific alterations of gut microbiota in patients with Membranous nephropathy: A Systematic Review and Meta-Analysis**

**Table S1** Literature retrieval strategy.

**Table S2** Details of gene analysis methods and fecal sample collection.

**Table S3** Quality assessment of selected studies using the Newcastle-Ottawa Scale.

**Table S4** Alpha diversity between case groups and control groups.

**Table S5** Beta diversity between case groups and control groups.

**Table S6** Alteration of gut microbiota at the phylum level.

**Table S7** Alteration of gut microbiota at the genus level.

**Table S8** Metabolites derived from gut microbiota.

Table S1. Search strategy

**PubMed**

Search number Query Results

4 ((((((microbio*[Title/Abstract]) OR (bacteria[Title/Abstract])) OR (dysbiosis[Title/Abstract])) OR (microflora[Title/Abstract])) OR (flora[Title/Abstract])) AND ((((((faecal[Title/Abstract]) OR (fecal[Title/Abstract])) OR (intestinal[Title/Abstract])) OR (gut[Title/Abstract])) OR (gastrointestinal[Title/Abstract])) OR (stool[Title/Abstract]))) AND (((((((((((((((((((((((membranous nephropathy) OR (Glomerulonephritis, Membranous)) OR (Membranous Glomerulonephritides)) OR (Membranous Glomerulonephritis)) OR (Nephropathy, Membranous)) OR (Membranous Glomerulopathy)) OR (Glomerulopathy, Membranous)) OR (Extramembranous Glomerulopathy)) OR (Glomerulopathy, Extramembranous)) OR (Membranous Glomerulonephropathy)) OR (Glomerulonephropathy, Membranous)) OR (Idiopathic Membranous Glomerulonephritis)) OR (Glomerulonephritides, Idiopathic Membranous)) OR (Glomerulonephritis, Idiopathic Membranous)) OR (Idiopathic Membranous Glomerulonephritides)) OR (Membranous Glomerulonephritides, Idiopathic)) OR (Membranous Glomerulonephritis, Idiopathic)) OR (Idiopathic Membranous Nephropathy)) OR(Membranous Nephropathy, Idiopathic)) OR (Nephropathy, Idiopathic Membranous)) OR (Heymann Nephritis)) OR (Nephritis, Heymann))) 8

3 ((((((((((((((((((((((membranous nephropathy) OR (Glomerulonephritis, Membranous)) OR (Membranous Glomerulonephritides)) OR (Membranous Glomerulonephritis)) OR (Nephropathy, Membranous)) OR (Membranous Glomerulopathy)) OR (Glomerulopathy, Membranous)) OR (Extramembranous Glomerulopathy)) OR (Glomerulopathy, Extramembranous)) OR (Membranous Glomerulonephropathy)) OR (Glomerulonephropathy, Membranous)) OR (Idiopathic Membranous Glomerulonephritis)) OR (Glomerulonephritides, Idiopathic Membranous)) OR (Glomerulonephritis, Idiopathic Membranous)) OR (Idiopathic Membranous Glomerulonephritides)) OR (Membranous Glomerulonephritides, Idiopathic)) OR (Membranous Glomerulonephritis, Idiopathic)) OR (Idiopathic Membranous Nephropathy)) OR(Membranous Nephropathy, Idiopathic)) OR (Nephropathy, Idiopathic Membranous)) OR (Heymann Nephritis)) OR (Nephritis, Heymann)) 7,876

2 ((((microbio*[Title/Abstract]) OR (bacteria[Title/Abstract])) OR (dysbiosis[Title/Abstract])) OR (microflora[Title/Abstract])) OR (flora[Title/Abstract]) 624,374

1 (((((faecal[Title/Abstract]) OR (fecal[Title/Abstract])) OR (intestinal[Title/Abstract])) OR (gut[Title/Abstract])) OR (gastrointestinal[Title/Abstract])) OR (stool[Title/Abstract]) 711,353

**Embase**

#6 #3 AND #4 AND #5 14

#5 'microbio*':ab,ti OR 'bacteria':ab,ti OR 'dysbiosis':ab,ti OR 'microflora':ab,ti OR 'flora':ab,ti 793,749

#4 'stool':ab,ti OR 'faecal':ab,ti OR 'fecal':ab,ti OR 'intestinal':ab,ti OR 'gut':ab,ti OR 'gastrointestinal':ab,ti 936,565

#3 #1 OR #2 11,260

#2 'glomerulonephritis, membranous':ab,ti OR 'membranous glomerulonephritides':ab,ti OR 'nephropathy, membranous':ab,ti OR 'membranous glomerulopathy':ab,ti OR 'glomerulopathy, membranous':ab,ti OR 'membranous nephropathy':ab,ti OR 'extramembranous glomerulopathy':ab,ti OR 'glomerulopathy, extramembranous':ab,ti OR 'membranous glomerulonephropathy':ab,ti OR 'glomerulonephropathy, membranous':ab,ti OR 'heymann nephritis':ab,ti OR 'nephritis, heymann':ab,ti OR 'idiopathic membranous glomerulonephritis':ab,ti OR 'glomerulonephritides, idiopathic membranous':ab,ti OR 'glomerulonephritis, idiopathic membranous':ab,ti OR 'idiopathic membranous glomerulonephritides':ab,ti OR 'membranous glomerulonephritides, idiopathic':ab,ti OR 'membranous glomerulonephritis, idiopathic':ab,ti OR 'idiopathic membranous nephropathy':ab,ti OR 'membranous nephropathy, idiopathic':ab,ti OR 'nephropathy, idiopathic membranous':ab,ti 7,144

#1 'membranous glomerulonephritis'/exp 9,534

**Web of science**

4 #1 AND #2 AND #3 46

3 TS=((((((((((((((((((((((((membranous nephropathy) OR (Glomerulonephritis, Membranous)) OR (Membranous Glomerulonephritides)) OR (Membranous Glomerulonephritis)) OR (Nephropathy, Membranous)) OR (Membranous Glomerulopathy)) OR (Glomerulopathy, Membranous)) OR (Extramembranous Glomerulopathy)) OR (Glomerulopathy, Extramembranous)) OR (Membranous Glomerulonephropathy)) OR (Glomerulonephropathy, Membranous)) OR (Idiopathic Membranous Glomerulonephritis)) OR (Glomerulonephritides, Idiopathic Membranous)) OR (Glomerulonephritis, Idiopathic Membranous)) OR (Idiopathic Membranous Glomerulonephritides)) OR (Membranous Glomerulonephritides, Idiopathic)) OR (Membranous Glomerulonephritis, Idiopathic)) OR (Idiopathic Membranous Nephropathy)) OR(Membranous Nephropathy, Idiopathic)) OR (Nephropathy, Idiopathic Membranous)) OR (Heymann Nephritis)) OR (Nephritis, Heymann)))) 8,049

2((((TS=(microbio*)) OR TS=(microflora)) OR TS=(bacteria)) OR TS=(dysbiosis)) OR TS=(flora) 990,468

1 (((((TS=(gut)) OR TS=(gastrointestinal)) OR TS=(intestinal)) OR TS=(faecal)) OR TS=(fecal)) OR TS=(stool) 813,015

**Cochrane library**

#1 MeSH descriptor: [Glomerulonephritis, Membranous] explode all trees 122

#2 (Membranous Glomerulonephritides):ab,ti,kw OR (Membranous Glomerulonephritis):ab,ti,kw OR (Nephropathy, Membranous):ab,ti,kw OR (Membranous Glomerulopathy):ab,ti,kw OR (Glomerulopathy, Membranous):ab,ti,kw OR (Membranous Nephropathy):ab,ti,kw OR (Extramembranous Glomerulopathy):ab,ti,kw OR (Glomerulopathy, Extramembranous):ab,ti,kw OR (Membranous Glomerulonephropathy):ab,ti,kw OR (Glomerulonephropathy, Membranous):ab,ti,kw OR (Heymann Nephritis):ab,ti,kw OR (Nephritis, Heymann):ab,ti,kw OR (Idiopathic Membranous Glomerulonephritis):ab,ti,kw OR (Glomerulonephritides, Idiopathic Membranous):ab,ti,kw OR (Glomerulonephritis, Idiopathic Membranous):ab,ti,kw OR (Idiopathic Membranous Glomerulonephritides):ab,ti,kw OR (Membranous Glomerulonephritides, Idiopathic):ab,ti,kw OR (Membranous Glomerulonephritis, Idiopathic):ab,ti,kw OR (Idiopathic Membranous Nephropathy):ab,ti,kw OR (Membranous Nephropathy, Idiopathic):ab,ti,kw OR (Nephropathy, Idiopathic Membranous):ab,ti,kw 432

#3 #1 OR #2 432

#4 (flora):ab,ti,kw OR (microflora):ab,ti,kw OR (dysbiosis):ab,ti,kw OR (microbio*):ab,ti,kw OR (bacteri*):ab,ti,kw 57973

#5 #3 AND #4 1

| Table S2. Details of gene analysis methods and fecal sample collection. | | | | | |
| --- | --- | --- | --- | --- | --- |
| **Study** | **Analysis methods** | **Samples** | **Sampling container** | **Manufacturer** | **Specimens protection** |
| Wei Yu et al.2020 | PCR and 16S rRNA gene sequence | Stool | Sterile tube | N/A | Frozen |
| Ruijuan Dong et al.2020 | 16S rRNA gene sequence | Stool | N/A | N/A | Frozen |
| Jun Zhang et al.2020 | 16S rRNA gene sequence | Stool | 50 mL conical tubes | N/A | Frozen |
| Rui Lang et al.2020 | 16S rRNA gene sequence | Stool | N/A | N/A | Frozen |
| Mengfei Li et al.2022 | 16S rRNA gene sequence | Stool | N/A | N/A | Frozen |

Abbreviations: PCR, polymerase chain reaction; N/A, not available.

Table S3. Quality assessment of selected studies using the Newcastle-Ottawa Scale.

| **Study** | **Selection** | | | | | **Comparability** | | **Ascertainment exposure** | | | **Score** |
| --- | --- | --- | --- | --- | --- | --- | --- | --- | --- | --- | --- |
|  | **1** | **2** | **3** | | **4** | **5A** | **5B** | **6** | **7** | **8** |  |
| Wei Yu et al.2020 | ***** | ***** |  | ***** | | ***** |  | ***** | ***** |  | 6* |
| Ruijuan Dong et al.2020 | ***** | ***** |  | ***** | | ***** |  | ***** | ***** |  | 6***** |
| Jun Zhang et al.2020 | ***** | ***** |  | ***** | | ***** |  |  | ***** |  | 5* |
| Rui Lang et al.2020 | ***** | ***** |  | ***** | | ***** |  | ***** | ***** |  | 6***** |
| Mengfei Li et al.2022 | ***** | ***** |  | ***** | | ***** |  | ***** | ***** |  | 6***** |

Table S4. Alpha diversity between case groups and control groups.

| **Study** | **Results description** | **Alpha-diversity Index** | **Conclusion** |
| --- | --- | --- | --- |
| Wei Yu et al.2020 | The bacterial richness and diversity of DKD were significantly higher than those of MN. | Ace/Chao1, Shannon/Simpson | Increase |
| Ruijuan Dong et al.2020 | No significant differences in community richness and diversity were observed between IgAN, MN, and the healthy control. | Ace/Chao1, Shannon/Simpson | Similar |
| Jun Zhang et al.2020 | INS patients had a lower diversity index of Chao1 compared with HC and CKD. | Chao1, PD whole tree | Decrease |
| Rui Lang et al.2020 | The alpha diversity index showed that intestinal flora differed between the IMN and healthy groups. | Ace, Shannon | Decrease |
| Mengfei Li et al.2022 | There was a significantly lower alpha diversity in IMN patients compared with the HC group. | Chao1/Sobs，Shannon/Simpson | Decrease |

Abbreviations: DKD, diabetic kidney disease; MN, membranous nephropathy; IgAN, immunoglobulin A nephropathy; INS, idiopathic nephrotic syndrome; HC, healthy controls; IMN, idiopathic membranous nephropathy; CKD, chronic kidney disease; PD whole tree, phylogenetic diversity whole tree index.

Table S5. Beta diversity between case groups and healthy controls.

| **Study** | **Results description** | **Beta-diversity Index** | **Conclusion** |
| --- | --- | --- | --- |
| Wei Yu et al.2020 | The significant difference in the gut microbiome in DKD and MN. | PCoA/NMDS | Distinct |
| Ruijuan Dong et al.2020 | The microbial composition of patients with IgAN and MN deviated from those of the HCs. | PCoA | Distinct |
| Jun Zhang et al.2020 | The unweighted UniFrac metric revealed a separation trend among HCs and INS. | PCoA | Distinct |
| Rui Lang et al.2020 | The comparison of multiple samples (beta diversity) also revealed differences in flora between IMN and healthy groups. | PCA, PCoA | Distinct |
| Mengfei Li et al.2022 | PCoA revealed that the microbiota composition of IMN patients and HCs was significantly different. | PCoA | Distinct |

Abbreviations: DKD, diabetic kidney disease; MN, membranous nephropathy; IgAN, immunoglobulin A nephropathy; HCs, healthy controls; INS, idiopathic nephrotic syndrome; IMN, idiopathic membranous nephropathy; PCoA, principal coordinate analysis; NMDS, nonmetric multidimensional scaling; PCA, Principal component analysis.

**Table S6. Alteration of gut microbiota at phylum level.**

| **Study** | **Higher abundant phylotypes** | **Lower abundant phylotypes** |
| --- | --- | --- |
| Wei Yu et al.2020 | Proteobacteria, Actinobacteria, Firmicutes/Bacteroidetes | Bacteroidetes |
| Ruijuan Dong et al.2020 | Proteobacteria | Synergistetes |
| Jun Zhang et al.2020 | Fusobacteria, Proteobacteria | Firmicutes |
| Rui Lang et al.2020 | Bacteroidetes | Firmicutes |
| Mengfei Li et al.2022 | Proteobacteria, Actinobacteria | Bacteroidetes, Firmicutes |

Table S7. Alteration of gut microbiota at the genus level.

| **Study** | **Higher abundant phylotypes** | **Lower abundant phylotypes** |
| --- | --- | --- |
| Wei Yu et al.2020 | Peptostreptococcaceae_incertae_sedis, Clostridium_sensu_stricto_1, Streptococcus, Veillonella, Haemophilus, Bifidobacterium, Lactococcus, and Faecalibacterium | N/A |
| Ruijuan Dong et al.2020 | Escherichia-Shigella, Streptococcus, Enterobacteriaceae_unclassified, Peptostreptococcaceae_incertae_sedis, and Enterococcus | Lachnospira, Lachnospiraceae_unclassified, Clostridium_sensu_stricto_1, and Veillonella |
| Jun Zhang et al.2020 | Parabacteroides, Providencia, and Myroides | Lachnospira, Roseburia, Megamonas, Megasphaera, Fusobacterium, and Akkermansia |
| Rui Lang et al.2020 | Prevotella-9, Megamonas | N/A |
| Mengfei Li et al.2022 | Citrobacter, Streptococcus, Enterobacter, Clostridium_innocuum_group, Erysipelatoclostridium | Faecalibacterium, Agathobacter, Bacteroides, Subdoligranulum, unclassified_f__Lachnospiraceae, Ruminococcus, Fusicatenibacter, Klebsiella, Lachnoclostridium, Alistipes, Lachnospiraceae_NC2004_group, Adlercreutzia, UCG-002, unclassified_f__Ruminococcaceae, UCG-005, Eubacterium_eligens_group, _Lachnospira |

Abbreviations: N/A, not available.

Table S8. Metabolites derived from gut microbiota in MN.

| **Study** | **Index** | **Conclusion** |
| --- | --- | --- |
| Wei Yu et al.2020 | ABC transporters | Interconversion of pentose/glucuronate and membrane transport in relation to ABC transporters and the phosphotransferase system were increased in MN. |
| Mengfei Li et al.2022 | Lipid metabolism | The pathways involved in bacterial invasion of epithelial cells (ko05100), Alpha-linolenic acid metabolism (ko00592), Staphylococcus aureus infection (ko05150), and Arachidonic acid metabolism (ko00590) were higher in the fecal microbiome of the IMN group. |

Abbreviations: MN, membranous nephropathy; IMN, idiopathic membranous nephropathy.
